# Ensuring enantiomeric consistency in FAPI radiopharmaceuticals: comparative analysis of (S)- and (S/R)-FAPI-46/-74 confirms pharmacological equivalence

**DOI:** 10.1186/s41181-026-00432-4

**Published:** 2026-02-28

**Authors:** Magdalena Staniszewska, Ralph Hübner, Camilla Locatelli, Douglas Howard, Matilde Forni, Francis Man, Bent Wilhelm Schoultz, Syed Nuruddin, Ingrid Sofie Norberg-Schulz Hagen, Sherly Mosessian, Ken Herrmann, Valeska von Kiedrowski, Katharina Lückerath

**Affiliations:** 1 Preclinical Theranostics, Department of Nuclear Medicine, University Duisburg-Essen and DKTK-Partner Site University Hospital Essen, Hufelandstr. 55, Essen, 45131 Germany; 2 GE Healthcare, Chalfont St Giles, UK; 3 GE Healthcare, Oslo, Norway; 4 Oslo Imaging and Therapy Laboratory - Norsk Medisinsk Syklotronsenter AS, University of Oslo, Oslo, Norway; 5 Sofie Biosciences Inc, Dulles, VA USA

**Keywords:** FAPI-46, FAPI-74, Enantiomers, Pharmacodynamics, Pharmacokinetics

## Abstract

**Background:**

Enantiomeric composition may impact binding and potentially performance of a drug product but is not routinely tested and minimum acceptance criteria are not always established. Some batches of the precursors FAPI-46 and FAPI-74 used clinically contain a mixture of (*S*)- and (*R*)-enantiomers. This study investigates the in vitro pharmacodynamic characteristics of different enantiomeric compositions of FAP-targeting radioligands FAPI-46 and FAPI-74 and in vivo pharmacokinetics of FAPI-46 in naïve mice.

**Results:**

(*S*)-, (*R*)-, and (*S/R*)-enantiomers of radiolabeled FAPI-46 and FAPI-74 were evaluated for stability in human serum, binding affinity, internalization, retention, and selectivity using HT1080-hFAP cells and HEK293-hCD26 cells. All enantiomeric compositions showed high stability in human serum with minimal serum protein adhesion. (*S*)- and (*S/R*)-enantiomers exhibited comparable binding, internalization, and retention characteristics. High selectivity for FAP over CD26/DPP4 was maintained. (*R*)-enantiomers showed no specific binding to FAP. Additionally, the same enantiomers of ^68^Ga-labeled FAPI-46 were evaluated by dynamic positron emission tomography (PET) imaging (0–60 min post-injection) and ex vivo organ biodistribution in naïve BALB/c mice (10, 30, and 60 min, or 4 h post-injection) to compare their pharmacokinetic profiles. PET imaging revealed nearly identical time-activity curves for the enantiomers of [^68^Ga] Ga-FAPI-46, indicating similar whole-body distribution within 1 h post-injection, with rapid renal clearance and minimal muscle uptake. Ex vivo organ biodistribution confirmed comparable pharmacokinetic profiles between enantiomers, with no significant differences in tissue distribution.

**Conclusion:**

These findings provide important validation that current clinical (*S/R*)-mixtures of FAPI-46/-74 exhibit pharmacokinetic and pharmacodynamic behavior comparable to pure (*S*)-enantiomers, supporting regulatory confidence and cross-trial reproducibility in the global FAPI-46/-74 imaging landscape.

**Supplementary Information:**

The online version contains supplementary material available at 10.1186/s41181-026-00432-4.

## Introduction

Enantiomers are non-superimposable mirror-image forms of a molecule that can exhibit significantly different biological activities and pharmacokinetics, thereby influencing critical parameters for drug performance and safety (Moein and Tran 2020; Smith 2009). Perhaps the most widely known example is thalidomide, where the (*R*)-enantiomer exhibited the desired sedative effects, while the (*S*)-enantiomer led to profound embryonic toxicity and teratogenic effects (Fabro et al. 1967). The relevance of chirality in radiopharmaceuticals is exemplified by [^99^mTc]ECD, where only one enantiomer efficiently crosses the blood–brain barrier, whereas its mirror image shows markedly inferior performance (Walovitch et al. 1989). Therefore, incorporating a detailed analysis of enantiomeric composition into the radioligand development pipeline is essential for several reasons, even when equivalence is ultimately demonstrated. First, the diagnostic or therapeutic effectiveness of radioligands may hinge on their enantiomeric purity; for instance, more effective binding/uptake of one enantiomer to the target could lead to improved imaging contrast and accuracy or enhanced therapeutic effects (Ding et al. 1997; Kobayashi et al. 2012). Second, variations in enantiomeric ratios can impact metabolism and clearance rates, which are critical factors for optimizing imaging and dosing protocols in clinical settings (Ding et al. 1993). Furthermore, regulatory agencies often favor compounds with well-characterized pharmacological profiles, including clear distinctions in efficacy between enantiomers (McVicker and O’Boyle 2024). These considerations underscore that the development of a radiopharmaceutical is a pharmaceutical undertaking that requires consideration of chirality, manufacturing consistency, pharmacokinetics (PK), pharmacodynamics (PD), and ultimately patient safety.

Some clinical trials (NCT05641896; NCT05262855) evaluating the diagnostic value of radiolabeled FAPI-46 and FAPI-74 utilize mixed enantiomeric compositions with 80% (*S*)-and 20% (*R*)-enantiomer of FAPI-46 and 84% (*S*)-and 16% (*R*)-enantiomer of FAPI-74. Following improvements in the manufacturing process and the implementation of chiral purity testing, the current product consists of a ≥ 95% (*S*)-enantiomer. Given the accelerated clinical adoption of FAPI-46 and FAPI-74, establishing that the clinical (*S/R*)-mixtures perform comparably to enantiomerically pure formulations is important to ensure data comparability, regulatory compliance, and patient safety. In this study, we compared the in vitro pharmacodynamic characteristics of the (*S*)-enantiomer, the (*R*)-enantiomer and the (*S/R*)-enantiomer mix of radiolabeled FAPI-46 and FAPI-74. Additionally, in vivo differences in the pharmacokinetic profiles of the same [^68^Ga]Ga-FAPI-46 enantiomeric compositions were evaluated in naïve mice. By demonstrating pharmacodynamic and pharmacokinetic comparability of (*S/R*)-mixtures and the pure (*S*)-form, this study indicates that data from existing multicenter FAPI-46 and FAPI-74 trials remain valid and provides regulatory confidence.

## Materials and methods

Method details for cultivation of HT1080 (wildtype), HT1080-hFAP (overexpression of human fibroblast activation protein; hFAP), and HEK293-hCD26 (overexpression of human CD26) cells, flow cytometry, and radiolabelling can be found in the supplementary methods.

## Radiolabeling

^68^Ga-labeling was used for both precursors, although ^18^F-labeled FAPI-74 is used in the current clinical study (ClinicalTrials.gov ID: NCT05641896), because the goal was to compare the properties of the individual enantiomers and the enantiomer mixtures, and FAPI-74 can be radiolabeled with either radionuclide, leading to comparable results in humans (Giesel et al. 2021). Different enantiomeric composition of FAPI-46 and FAPI-74 were labelled with gallium-68 according to standard methods. The compounds analyzed were: (*S*)-, (*R*)-, (*S/R* = *80:20*)-enantiomers for [^68^Ga]Ga-FAPI-46 (named [^68^Ga]-FAPI-46 in the clinical trial NCT05262855) and (*S*)-, (*R*)-, (*S/R* = *84:16*)-enantiomers for [^68^Ga]Ga-FAPI-74 (named [^68^Ga]-FAPI-74 in the clinical trial NCT05641896). For the in vitro experiments, radiochemical purity and non-optimized molar activities were 95–98% and 24–33 MBq/nmol for [^68^Ga]Ga-FAPI-46 compounds, and > 98% and 7.3 MBq/nmol for [^68^Ga]Ga-FAPI-74 compounds. For the in vivo experiments, the radiochemical purity of [^68^Ga]Ga-FAPI-46 compounds at the end of synthesis was 95% and remained stable at 94–95% up to 4–5 h post-synthesis.

## Stability in human serum and adhesion to serum proteins

[^68^Ga]GaFAPI-46 compounds were passed through a C18 cartridge (Sep-Pak C18 Plus Short Cartridge, 360 mg Sorbent per Cartridge, 55–105 µm), which was preconditioned with 4 mL ethanol and 10 mL Tracepur water and dried with 10 mL air. The products were washed with 10 mL Tracepure water, eluted with 1000 μL ethanol/Tracepur water (9/1, v/v), and diluted with 9 mL 0.9% NaCl solution to yield a final ethanol concentration of < 10%. Radioligand solutions (7 × 100 μL) were added to 7 × 100 μL human serum and incubated at 37 °C. At predefined time points (10, 30, 60, 90, 120, 180, 240 min), 150 μL ethanol were added, and the precipitation of serum proteins was supported by cooling on ice for 2 min. After centrifugation at ~ 16,000 × *g* for 2 min at 4 °C, the supernatant was analyzed by analytical radio-HPLC (0–100% MeCN + 0.1% TFA in 10 min).

[^68^Ga]GaFAPI-74 compounds were diluted with 5 mL Tracepure water and passed through a cartridge (Oasis Plus light HLB), which was preconditioned with 4 mL ethanol and 10 mL Tracepur water and dried with 10 mL air. The products were washed with 5 mL Tracepure water, eluted with 1000 μL ethanol/Tracepur water (9/1, v/v), and diluted with 9 mL 0.9% NaCl solution to yield a final ethanol concentration of < 10%. Radioligand solutions (7 × 100 μL) were added to 7 × 100 μL human serum and incubated at 37 °C. At defined time points (10, 30, 60, 90, 120, 180, 240 min), the mixtures were cooled on ice for 2 min. After centrifugation at ~ 16,000 × *g* for 2 min at 4 °C, the supernatant was analyzed by analytical radio-HPLC (0–100% MeCN + 0.1% TFA in 10 min).

## Binding, internalization, retention and selectivity

A schematic illustrating the experimental workflow for the assays described below is shown in Suppl. Figure 3. Cells (10^6^ cells/sample in DMEM/5% BSA; triplicate samples) were transferred to low binding reaction tubes. After addition of 1 kBq (peptide:target = 1:2) [^68^Ga]Ga-FAPI-46 or [^68^Ga]Ga-FAPI-74 in different enantiomeric compositions, samples were incubated for 1 h and 4 h at 37 °C/5% CO_2_. Cells were centrifuged at 350 × g for 5 min at 4 °C and the supernatant and pipette tips were collected (unbound fraction). Cells were washed with 500 µL PBS pH 7.4 (350 × g, 5 min, 4 °C) and the wash solution and pipette tips were collected (unbound fraction). The cell pellet was incubated with 300 µL glycine–HCl pH 2.8 for 1 min and the supernatant and pipette tips were collected (membrane-bound fraction). Cells were washed with 500 µL PBS pH 7.4 (350 × g, 5 min, 4 °C) and the wash solution and pipette tips were collected (membrane-bound fraction). The remaining cell pellet in the reaction tube (internalized fraction) was transferred to a gamma-counter tube. To determine radioligand retention, cells were incubated with radioligands as above for 1 h. The radioactive media was removed, and cells were washed with PBS; this yielded the initially cell-associated activity. Fresh non-radioactive culture media was added, and cells were incubated for another hour at 37 °C/5%CO_2_ before unbound, membrane-bound and internalized fractions were collected. All collected fractions were measured in a gamma-counter (Perkin145 Elmer Gamma Counter 2480 Wizard^2^) to quantify the overall cell-associated and internalized radioactivity. Data were background- and decay-corrected and expressed as percent added activity per 10^6^ cells (% AA/10^6^ cells). Competition studies were performed as above but included the addition of the respective cold *(S)*-enantiomer as competitor in 10 increasing concentrations ranging from 0.001 to 1000 nM, whereas the eleventh well contained no competitor to ensure unimpeded binding of the radioligand. The half-maximal inhibitory concentration (IC_50_) was calculated by fitting the data using nonlinear regression analysis.

## In vivo positron-emission tomography (PET) imaging and organ biodistribution

Detailed descriptions of the imaging and biodistribution procedures are provided in the supplementary methods. Briefly, in vivo PET imaging and ex vivo organ biodistribution were conducted for the (*S*)-, (*R*)- and (*S/R*)-enantiomers of [^68^Ga]GaFAPI-46 (n = 12 per enantiomer) in male BALB/cJRj mice, each receiving an intravenous bolus of 4–5 MBq [^68^Ga]GaFAPI-46 in 100–200 µL (the catheter was then flushed with 100 μL saline to remove residual radioactivity left in the catheter after injection). Twenty-five organs of interest were collected at 10, 30, 60, or 240 min post-injection for gamma-counting (n = 3 per enantiomer group and timepoint). Measurements were performed using a Hidex AMG automatic gamma counter (480–558 keV window), with decay correction, normalization, and detector efficiency calibration applied to calculate the percent injected activity per gram tissue (%IA/g). Mice in the 240-min cohort underwent 60-min dynamic whole-body PET/CT imaging (MR Solutions Benchtop) under isoflurane anesthesia (4% induction, 2.0–2.5% maintenance in 100% O_2_) with temperature control, followed by euthanasia and biodistribution analysis. Reconstructed PET images were analyzed using Imalytics software (version 3.1.1.0) to determine the %IA/g, with volumes of interest defined for major organs based on CT segmentation and bladder activity identified on the summed PET image.

## Statistics

Data are presented as mean and standard deviation (SD) of independent experiments. To support conclusions regarding comparability, binding characteristics of (*S*)- and (*S/R*)-enantiomeric compositions of [^68^Ga]Ga-FAPI-46 and [^68^Ga]Ga-FAPI-74, respectively, on HT1080-hFAP cells were compared using the the Mann Whitney test. Organ retention of [^68^Ga]Ga-FAPI-46 enantiomeric compositions were analyzed based on the ex vivo biodistribution data using one-way-ANOVA with Tukey’s post hoc test for multiple comparisons. Statistical significance was set to < 0.05. Analyses were performed in GraphPad Prism version 10.

## Results

### Stability in human serum is comparable for different enantiomeric compositions of [^68^Ga]Ga-FAPI-46 and [^68^Ga]Ga-FAPI-74

**Fig. 1:**
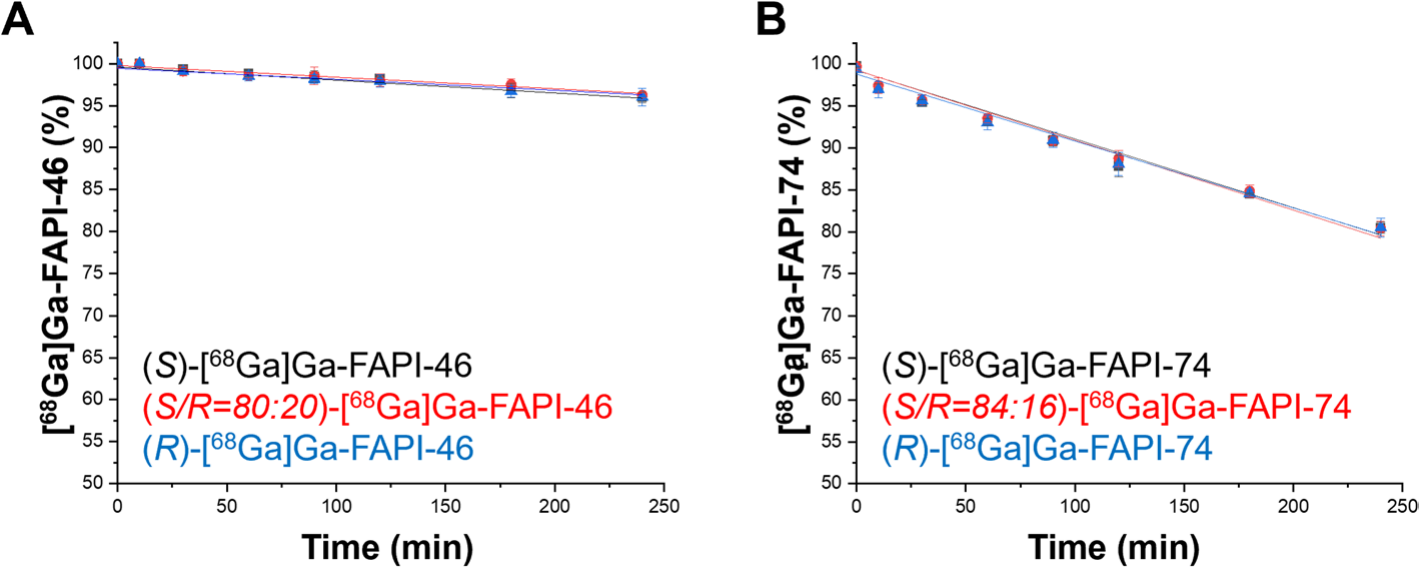
Stability of different enantiomeric compositions of [^68^Ga]Ga-FAPI in human serum. Mean ± SD of the fraction of intact [^68^Ga]Ga-FAPI-46 (**A**; n = 4) and [^68^Ga]Ga-FAPI-74 (**B**; n = 3)

All enantiomeric compositions of [^68^Ga]Ga-FAPI-46 and [^68^Ga]Ga-FAPI-74 were stable in human serum (at 240 min: [^68^Ga]Ga-FAPI-46 > 96%, [^68^Ga]Ga-FAPI-74 > 80%) (Fig. 1, Suppl. Figs. 1, 2). Adhesion to serum proteins was > 5%, indicating that differences in plasma protein binding are unlikely to contribute to the observed PK/PD behavior (Suppl. Tables 1, 2).

### (***S***)- and (***S/R***)-enantiomers of [^68^Ga]Ga-FAPI compounds exhibit comparable binding, internalization and retention characteristics

**Fig. 2:**
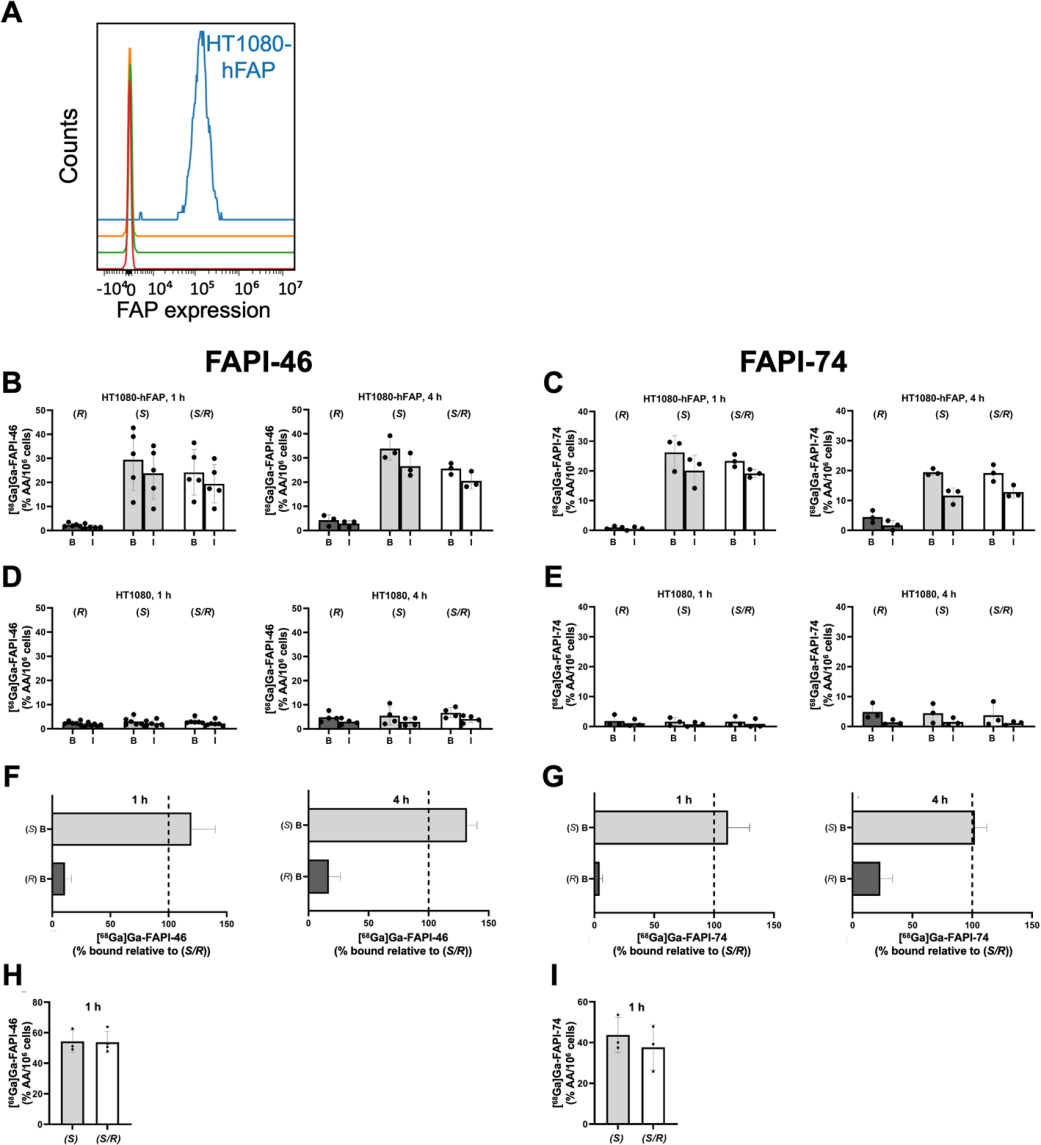
Binding characteristics of different enantiomeric compositions of [^68^Ga]Ga-FAPI compounds. A schematic illustrating the experimental workflow is shown in Suppl Fig. 3. **A** Flow cytometric quantification of FAP expression in HT1080 and HT1080-hFAP cells (unstained vs. stained). FAP expression is only detected in HT1080-hFAP cells. One representative experiment out of two is shown. **B–E** HT1080-hFAP and HT1080 cells were incubated with (*S*)-, (*S/R*)-, or (*R*)-[^68^Ga]Ga-FAPI-46 or -[^68^Ga]Ga-FAPI-74 for 1 h or 4 h. Overall cell-associated and internalized fractions of [^68^Ga]Ga-FAPI-46/-74 were determined. **B**,** C** HT1080-hFAP ([^68^Ga]Ga-FAPI-46: 1 h, n = 5, 4 h, n = 3; [^68^Ga]Ga-FAPI-74: n = 3). Differences between the (*S*)- and (*S/R*)-entantiomeric compositions of FAPI-46 (B) and FAPI-74 (C), respectively, were not statistically significant (*p* ≥ 0.1000). **D**, **E** HT1080 ([^68^Ga]Ga-FAPI-46, n = 6; [^68^Ga]Ga-FAPI-74, n = 3). **F**, **G** Binding of the (*S)*- and (*R)*-enantiomers to HT1080-hFAP was normalized to that of *(S/R)*-[^68^Ga]Ga-FAPI-46/−74. **H**, **I** Retention. After incubation with radioligands for 1 h, cells were washed, incubated with non-radioactive medium for 1 h, and internalized fractions were analyzed ([^68^Ga]Ga-FAPI-46, n = 4; [^68^Ga]Ga-FAPI-74, n = 3). Differences between the (*S*)- and (*S/R*)-entantiomeric compositions of FAPI-46 (H) and FAPI-74 (I), respectively, were not statistically significant (*p* ≥ 0.1143). Columns and bars represent the mean ± SD of the %AA/10^6^ cells; individual data points represent independent experiments as the average of triplicate samples. B—bound, overall cell-associated fraction, I—internalized fraction

To validate our experimental cell system, we quantified the expression of FAP on the tumor cell surface. HT1080-hFAP cells expressed 314.905 ± 15.376 FAP molecules/cell while no expression was detectable in HT1080 cells (Fig. 2A).

For both FAPI-46 and FAPI-74, *(S)*-[^68^Ga]Ga-FAPI and *(S/R)*-[^68^Ga]Ga-FAPI bound to hFAP on HT1080-hFAP cells and were internalized (~ 80%), whereas *(R)*-[^68^ Ga]Ga-FAPI exhibited no specific binding to HT1080-hFAP cells. Binding to HT1080 wildtype cells was negligible for all enantiomers (Fig. 2B–D, Suppl. Tables 3, 4). *(S)*-[^68^Ga]Ga-FAPI and *(S/R)*-[^68^Ga]Ga-FAPI demonstrated comparable binding, with slightly enhanced binding for the (*S*)-enantiomer, which is in line with the presence of non-binding (*R*)-enantiomer in *(S/R)*-[^68^Ga]Ga-FAPI (Fig. 2F,G). In agreement with these data, the IC_50_ were 8.9 ± 1.6 nM for *(S)*-[^68^Ga]Ga-FAPI-46 and 15.2 ± 1.9 nM for *(S/R)*-[^68^Ga]Ga-FAPI-46 (n = 4), and 43.0 ± 4.7 nM for *(S)*-[^68^Ga]Ga-FAPI-74 and 42.31 ± 5.34 nM for *(S/R)*-[^68^Ga]Ga-FAPI-74 (n = 5).

Finally, when assessing cellular retention/efflux, ~ 50% of the initially cell-associated fraction of *(S)*-[^68^Ga]Ga-FAPI-46 and *(S/R)*-[^68^Ga]Ga-FAPI-46 and ~ 44% and ~ 35% of *(S)*-[^68^Ga]Ga-FAPI-74 and *(S/R)*-[^68^ Ga]Ga-FAPI-74 remained internalized in HT1080-hFAP cells at 1 h after radioligand removal from the media (Fig. 2H, I,Suppl. Tables 5, 6).

### Different enantiomeric compositions of [^68^Ga]Ga-FAPI compounds maintain selectivity for hFAP

**Fig. 3:**
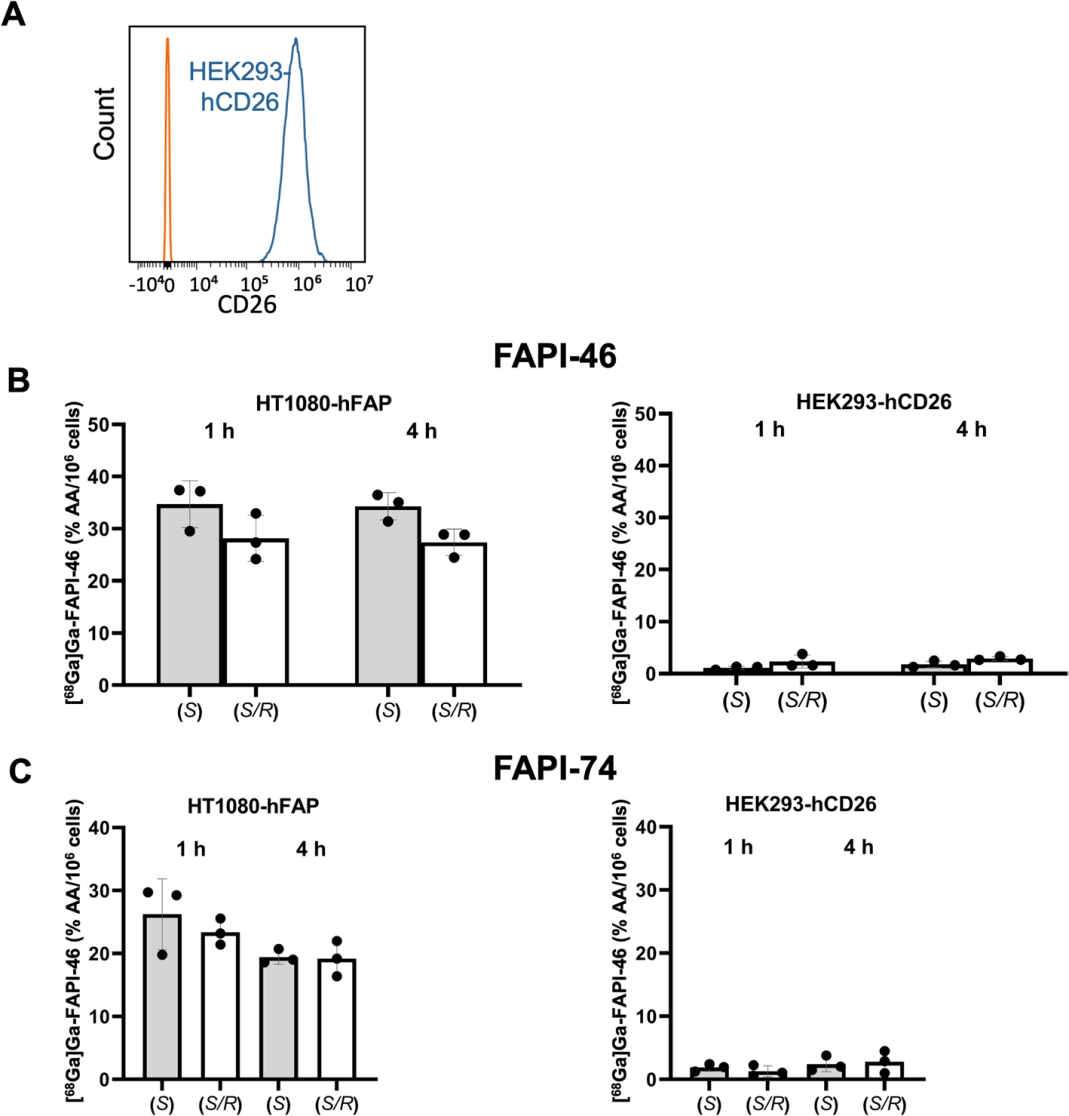
Selectivity.** A** Flow cytometric quantification of CD26/DPP4 expression in HEK293-hCD26 cells (blue, stained with anti-hCD26-APC; orange—unstained). One representative experiment out of two is shown. **B**,** C** HT1080-hFAP and HEK293-hCD26 cells were incubated with (*S*)- or (*S/R*)-[^68^Ga]Ga-FAPI-46 (**B**) or (*S*)- or (*S/R*)-[^68^Ga]Ga-FAPI-74 (**C**) for 1 h or 4 h, and overall cell-associated and internalized fractions were determined (n = 3). Columns and bars represent the mean ± SD of the %AA/10^6^ cells; individual data points represent independent experiments as the average of triplicate samples

Selectivity of [^68^Ga]Ga-FAPI compounds was tested using HEK293-hCD26 cells, which express hCD26/hDPP4 (~ 650.000 CD26/cell), a protein with high sequence similarity to hFAP (Fig. 3A). (*S*)- and (*S/R*)-[^68^Ga]Ga-FAPI compounds did not bind to HEK293-hCD26 over background levels, whereas their binding to and internalization in HT1080-hFAP cells was confirmed (Fig. 3B, C).

### (***S***)-, (***S/R***)***-***, and (***R***)***-*** enantiomers of [^68^Ga]Ga-FAPI-46 exhibit comparable in vivo pharmacokinetic characteristics

**Fig. 4:**
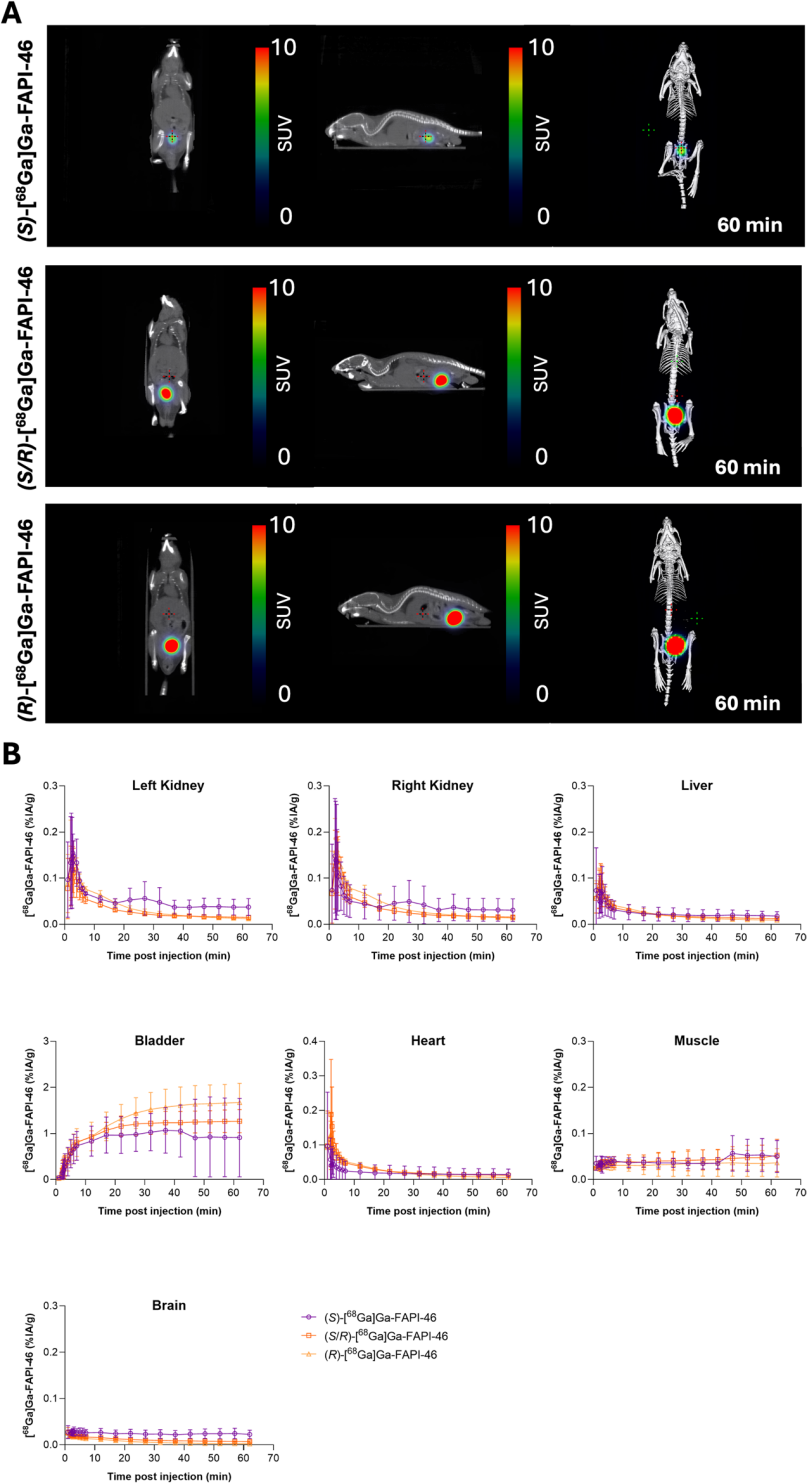
In vivo PET imaging.** A** Representative PET/CT images (60 min post-injection) of naive mice administered (*S*)-, (*S/R*)- or (*R*)-[^68^Ga]Ga-FAPI-46 and dynamically imaged over 60 min (one representative mouse out of three per enantiomer is shown). The left panel displays a coronal view, the middle panel shows a sagittal view, and the right panel presents a maximum intensity projection (MIP) of the skeletal structure. The color scale bar represents standardized uptake values (SUV) ranging from 0 to 10, with regions of higher uptake shown in red and lower uptake in blue. PET images are displayed using identical SUV scaling across all enantiomeric compositions. High renal and bladder activity dominates the visual signal in naïve mice; quantitative comparison of tissue uptake was therefore based on time-activity curves and *ex vivo* biodistribution data. BL, urinary bladder; H, heart; K, kidneys; Cor., coronal plane; Sag., sagittal plane. **B** Time-activity curves of (*S*)-, (*R*)- and (*S/R*)-[^68^Ga]Ga-FAPI-46 uptake in organs, based on dynamic PET/CT imaging. All [^68^Ga]Ga-FAPI-46 enantiomers showed comparable results. For A and B: n = 3 mice/enantiomer

The distribution profile of *(S)*-, (*R*)- and *(S/R)*-[^68^Ga]Ga-FAPI-46 was evaluated by PET imaging and ex vivo biodistribution in healthy mice. Naïve mice were chosen to isolate PK effects attributable solely to stereochemistry. PET imaging showed that all enantiomeric compounds distributed rapidly and similarly throughout the body within the first 5 min post-injection, with clearance via the urinary tract evident by 60 min. Radioactivity was observed in the heart and large abdominal vessels in the first 2 min post-injection, followed by rapid accumulation in the kidneys and bladder. By ~ 5 min post-injection, the activity was predominantly localized in the bladder, with minimal residual signal in the kidneys. No significant uptake was observed in non-excretory organs in naïve mice (Fig. 4).

**Fig. 5:**
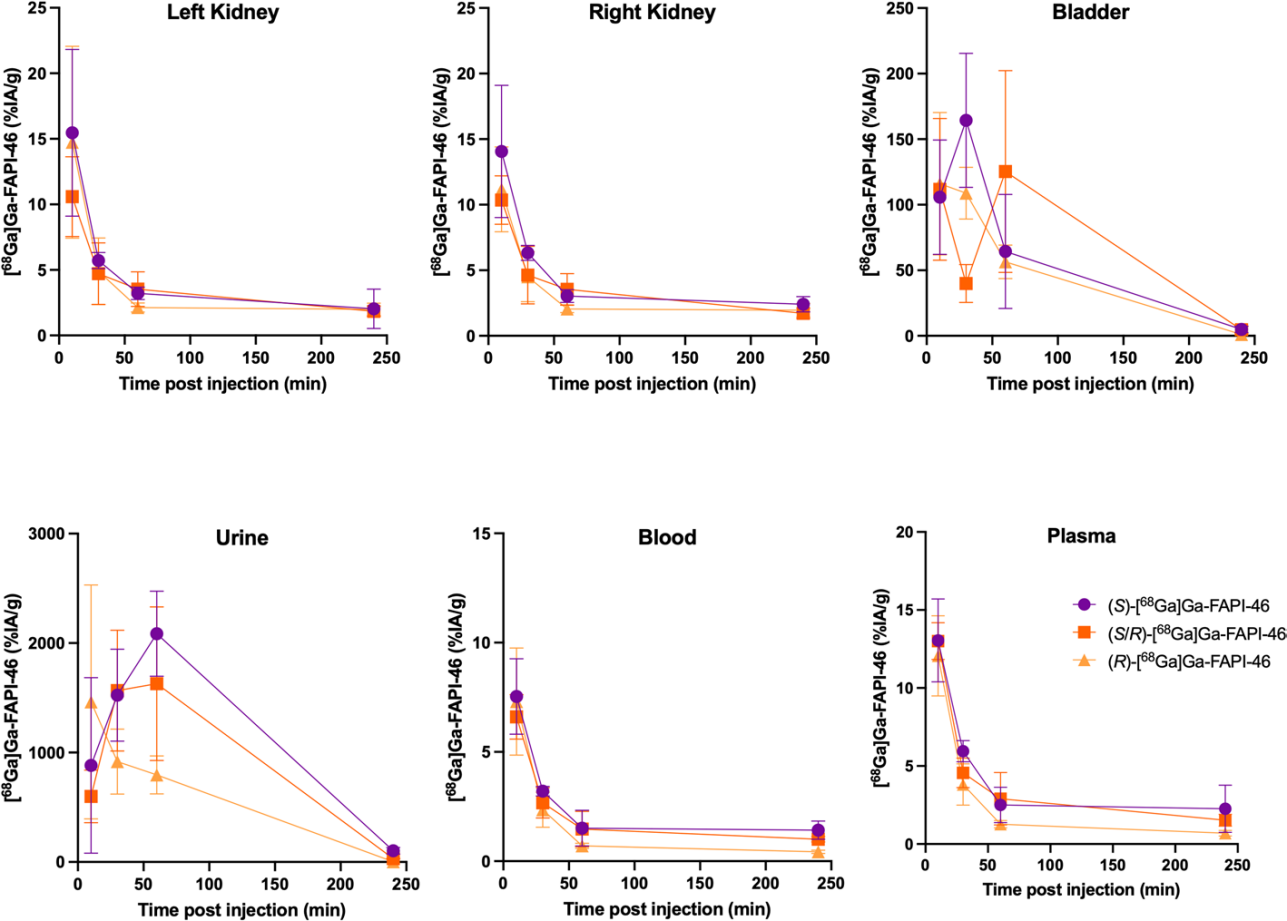
Ex vivo biodistribution of (S)-, (R)-, and (S/R)-[^68^Ga]Ga-FAPI-46 in naïve mice over 4 h in selected organs. Radioactivity levels (%IA/g) were measured in selected tissues and fluids including left kidney, right kidney, bladder, urine, whole blood, and plasma at 10, 30, 60, and 240 min post-injection. Each mouse received an intravenous bolus of 4–5 MBq (~ 0.33–0.45 µg of FAPI-46 per mouse) of the respective radioligand. Biodistribution was assessed by gamma counting following euthanasia. Data are presented as mean ± standard deviation (n = 3 mice per enantiomer and time point). [^68^Ga]Ga-FAPI-46 enantiomers showed comparable results

The ex vivo biodistribution results of (*S*)-, (*R*)-, and (*S/R*)-[^68^Ga]Ga-FAPI-46 confirmed the PET/CT imaging results, showing rapid clearance of all three compounds from the blood through the kidneys and into the urinary bladder (Fig. 5, Suppl. Figs 4–7, Suppl. Tables 7–9). Overall, PET/CT imaging and gamma-counting data showed no discernible differences in distribution profiles between the enantiomers.

## Discussion

Systematic evaluation of chirality and its impact on PK/PD behavior is a foundational pharmaceutical principle that is increasingly necessary in radiopharmaceutical development; it helps to address a gap that has historically been underappreciated in nuclear medicine, where empirical application has at times outpaced pharmaceutical rigor. The present study addresses this translational need by providing a comprehensive assessment of the influence of enantiomeric composition on the performance of FAPI-46 and FAPI-74. Our findings demonstrate that the (*S*)-enantiomers and the currently used (*S/R*)-enantiomer mixtures, but not the (*R*)-enantiomer, exhibit comparable binding, internalization, and retention properties, while maintaining high selectivity for FAP over the structurally related protein CD26/DPP4. In line with these observations, thein vivo results suggest no significant differences between the PK properties of the (*S*)-, (*S/R*)- and (*R*)-enantiomers. As FAPI-46 and FAPI-74 imaging expands globally, our data may provide an empirical foundation relevant to regulatory assessment of mixed-enantiomer batches. By demonstrating PK/PD equivalence with respect to the tested properties, this study indicates that the extensive clinical data acquired with current (*S/R*)-FAPI-46/74- formulations remain valid and comparable to future enantiomerically pure products.

The (*S*)-enantiomers of both FAPI-46 and FAPI-74 are responsible for the specific binding to FAP, while the (*R*)-enantiomers exhibit negligible binding. This finding aligns with previous studies on other chiral compounds where specific enantiomers often demonstrate superior target engagement (Moein and Tran 2020; Smith 2009). For example, a study on DPP4 ligands with structural similarity to FAPI-46 and FAPI-74 showed that the chiral center bearing the nitrile group was critical for the DPP4-inhibitory activity of these compounds (Hughes et al. 1999). Interestingly, the (*S/R*)-mixtures of FAPI-46 and FAPI-74 currently used in clinical investigations showed only moderately reduced binding compared to the pure (*S*)-enantiomers. This suggests that the presence of the non-binding (*R*)-enantiomer in the current mixture does not significantly impair overall target engagement, likely due to the high binding affinity of the (*S*)-enantiomer. While the use of pure (*S*)-enantiomers could enhance diagnostic performance, the small difference in active compound between the (*S*)- and (*S/R*)-batches suggests that the presence of the (*R*)-enantiomer is unlikely to have a meaningful impact on imaging accuracy in current clinical studies. However, as the field of precision medicine advances, even marginal improvements in targeting efficiency could become clinically relevant, especially for therapeutic applications where optimizing the therapeutic index is crucial (U.S. 1992; Agency 1993).

The maintained selectivity of all tested FAPI compositions for FAP over CD26/DPP4 is noteworthy. This high selectivity is crucial for minimizing off-target effects and potential toxicities, which is a key consideration in the development of targeted radiopharmaceuticals.

The highly similar in vivo PK profile of different enantiomeric compositions of [^68^Ga]Ga-FAPI-46 in mice, along with the comparable stability of enantiomers of both FAPI-46 and FAPI-74 in human serum and their low adhesion to serum proteins suggest that the clinical PK/PD profiles of the (*S*)- and (*S*/*R*)-compounds may be similar. These findings could have direct implications for clinical translation, as they suggest that existing dosing and imaging protocols developed for the (*S/R*)-FAPI-46/-74 mixtures likely remain valid if a transition to pure (*S*)-FAPI-46/-74 enantiomers is considered in the future. From a translational perspective, the observed similarity in PK/PD behavior between enantiomerically pure (*S*)-FAPI-46/-74 and clinically used (*S/R*)-mixtures supports the comparability of data across studies employing precursors with controlled differences in enantiomeric composition. Consistent with regulatory guidance on stereoisomeric drugs (U.S. 1992; Agency 1993), these findings indicate that, when the non-active enantiomer does not measurably affect critical quality attributes or biological performance, limited variability in enantiomeric purity may be scientifically justified. At the tested molar activities, such variability is unlikely to impose additional constraints on specific activity requirements for diagnostic applications, while warranting continued evaluation in therapeutic settings.

In addition, FDA and EMA guidance emphasize that the stereoisomeric composition of drug substances should be clearly characterized and appropriately controlled, supported by defined stereochemical identity and purity specifications as well as stereoselective analytical methods where necessary (U.S. 1992; Agency 1993). Manufacturing processes should ensure reproducible stereoisomeric composition within specification, stability programs should incorporate methods capable of assessing stereochemical integrity, and quantitative evaluation of individual enantiomers in vivo is recommended early in development. These principles are particularly relevant for radiopharmaceuticals, where the radiolabeled active substance administered to patients may not always be readily isolable or fully characterizable, and quality assurance therefore relies heavily on upstream control of the nonradioactive precursor and the manufacturing process. Accordingly, enantiomeric composition should be monitored as a critical quality attribute to minimize batch-to-batch variability in the fraction of the pharmacologically active enantiomer. Importantly, the objective is to ensure robust process control rather than to maximize enantiomeric purity in every instance. When a defined enantiomeric mixture is used, its composition should be specified and justified based on pharmacological and safety considerations. Consistent with these principles, our data demonstrate comparable in vitro PD behavior for the (*S*) and (*S/R*) compositions—together with absent specific binding of the (*R*) form—and similar in vivo distribution and clearance profiles for [^68^Ga]Ga-FAPI-46 in naïve mice, supporting the conclusion that controlled (*S/R*) mixtures can be pharmacologically comparable at tracer doses.

Limitations of this study include that in vivo experiments were performed exclusively in naïve mice in order to isolate stereochemistry-dependent PK effects from confounding influences that occur in commonly used FAP-overexpressing xenograft models, which often have supraphysiological and homogenous FAP expression levels (which are unlikely to reveal subtle differences in ligand affinity or PK and, therefore, may not accurately reflect clinically relevant performance distinctions); however, this design does not allow assessment of potential stereoselective differences in tumour uptake, retention, or tumour-to-background contrast. Furthermore, in vivo investigations were conducted using ^68^Ga-labeled compounds and restricted to FAPI-46. To date, FAPI-46 is the most extensively characterized and clinically applied FAPI radioligand and therefore provides a robust reference for evaluating potential stereochemistry-dependent effects on whole-body PK. FAPI-46 and FAPI-74 share the same FAP-binding motif and chiral center, and the concordant in vitro results observed for both compounds indicate that stereochemical effects on target binding and cellular handling are conserved across these analogues. Accordingly, in vivo assessment of FAPI-46 was considered sufficient to interrogate stereochemistry-driven differences in systemic distribution while avoiding unnecessary duplication of animal experiments. While FAPI-74 has demonstrated comparable clinical performance when labeled with either ^68^Ga or ^18^F, radionuclide-specific coordination chemistry (^18^F-AlF labeling) may theoretically influence PK or retention and should be addressed in future radionuclide-specific studies. Based on the fast PK of FAPI-46 and FAPI-74, the in vivo evaluation was limited to early time points (≤ 4 h post-injection), such that potential effects on late redistribution or long-term retention could not be addressed. Finally, as with all preclinical studies, interspecies differences in FAP expression, radioligand metabolism, and clearance may limit direct extrapolation to human PK, underscoring the importance of continued clinical validation.

## Conclusions

This work supports the pharmacokinetic and pharmacodynamic comparability of clinically used (*S/R*)-FAPI-46 and FAPI-74 radioligands and their pure (*S*)-forms, providing important assurance for ongoing and future multicenter studies, and regulatory submissions while also highlighting the potential for future optimization through the use of pure (*S*)-enantiomers. This work underscores the importance of considering enantiomeric compositions in the development of targeted radiopharmaceuticals and provides a foundation for further investigations aimed at refining these promising diagnostic and therapeutic agents.

## Supplementary Information

Additional file1

## Data Availability

The datasets generated during the current study are available from the corresponding author on reasonable request.
