# Supplementary material for: Ensuring enantiomeric consistency in FAPI radiopharmaceuticals: comparative analysis of (S)- and (S/R)-FAPI-46/-74 confirms pharmacological equivalence
Source: EJNMMI Radiopharm Chem. 2026 Feb 28;11:23. doi: 10.1186/s41181-026-00432-4 (PMC13057127; doi:10.1186/s41181-026-00432-4)
Supplement: Supplementary file 1 — Additional file1 [file 41181_2026_432_MOESM1_ESM.docx]

**Supplementary materials - Impact of different enantiomeric compositions of FAPI-46 and FAPI-74 on *in vitro* pharmacodynamic and *in vivo* pharmacokinetic characteristics**

Madalena Staniszewska^1*^, Ralph Hübner^1*^, Camilla Locatelli^1^, Douglas Howard^1^, Matilde Forni^2^, Francis Man^2^, Bent Wilhelm Schoultz^3^, Syed Nuruddin^3^, Ingrid Sofie Norberg-Schulz Hagen^3^, Sherly Mosessian^4^, Ken Herrmann^1^, Valeska von Kiedrowski^1^, Katharina Lückerath^1#^

^1^ University Duisburg-Essen and DKTK-partner site University Hospital Essen, Department of Nuclear Medicine, Essen, Germany

^2^ GE Healthcare, Chalfont St Giles, UK, and Oslo, Norway

^3^ Oslo Imaging and Therapy Laboratory - Norsk Medisinsk Syklotronsenter AS, University of Oslo, Norway

^4^ SOFIE, Dulles, Virginia, USA

^*^equal contribution

^#^corresponding author

**Supplementary Methods**

**Cells**

HT1080 cells expressing human fibroblast activation protein (hFAP; HT1080-hFAP), HEK293 cells expressing hCD26 (hDPP4; both, Uwe Haberkorn, University Hospital Heidelberg, Germany), and HT1080 cells (Diana Klein, University Hospital Essen, Germany) were cultivated in Dulbecco Modified Eagle Medium (DMEM)/5% fetal bovine serum at 37°C/5% CO_2_. Absence of contamination with mycoplasma was ensured regularly.

**Flow cytometry**

FAP and CD26 expression were quantified using an anti-hFAP-AlexaFluor^®^647 antibody (1:50, clone 427819, R&D Systems #FAB3715R) and an anti-CD26-PE antibody (1:50, clone FR10-11G9, Miltenyi #130-126-411), respectively according to the manufacturer’s instructions. FAP receptor quantity was determined using Quantum™ SimplyCellular® anti-Mouse IgG quantification beads (Polysciences #BLI815B-5) according to the manufacturer’s instructions. Samples were measured with a CytoFlex S flow cytometer (Beckman Coulter) and analyzed using FlowJo software (Tri Star Inc).

**Radiolabeling**

*In vitro* experiments: Different enantiomeric compositions of FAPI-46 and FAPI-74 were labelled with gallium-68. Precursors (1 mg/mL Tracepur water; FAPI-46, 7.5 μg; FAPI-74, 18-25 µg) were added to ^68^Ga^3+^ (FAPI-46, 200-280 MBq, FAPI-74, 180-250 MBq) in a solution obtained by fractioned elution of a ^68^Ge/^68^Ga generator (IGG100, Eckert and Ziegler) with HCl (0.1 M, 1.0 mL) and subsequent titration to pH 3.6-4.2 by addition of sodium acetate solution (FAPI-46, 1.25 M, 125-150 μL; FAPI-74, 2 M, 200 µL) and 50 µL ethanol (FAPI-74 only). After reaction for 10 min at 99°C (FAPI-46) and 5 min at ambient temperature (FAPI-74), respectively, the reaction mixtures were analyzed by analytical radio-HPLC (Chromolith^®^ HighResolution RP-18, endcapped, 100-4,6, L × I.D. 100 mm x 4.6 mm HPLC column).

*In vivo* experiments: For (*S*)-[⁶⁸Ga]Ga-FAPI-46 and (*S/R*)-[⁶⁸Ga]Ga-FAPI-46, 100 µL MilliQ water followed by 450 µL of 1.5 M sodium acetate (pH 9.0) and 350 µL of 0.07 M sodium ascorbate were added directly to the precursor vial (50 µg). The precursor solution was transferred to a new 10 mL glass reaction vial. For (*R*)-[⁶⁸Ga]Ga-FAPI-46, 1 mL MilliQ water was added directly to the precursor vial (1 mg), resulting in a precursor stock solution of 1 mg/mL. From this stock, 50 µL (50 µg precursor) was transferred directly into a 10 mL glass reaction vial and diluted with additional 50 µL MilliQ water, 450 µL 1.5 M sodium acetate (pH 9.0) and 350 µL 0.07 M sodium ascorbate. Radiolabeling of the enantiomers was done by automated elution of [^68^Ga]Ga^3+^ in 0.1 M HCl (5.0 mL) from a ^68^Ge/^68^Ga generator. The elution was collected directly into the reaction vial and the date and time of elution as well as radioactivity at end of elution was recorded. Following elution, the reaction vial was placed in a pre-heated heating block (95 ± 2 ºC) for 10 min. The reaction vial was cooled for ~5 min, and the pH of the final product was measured with both electronic pH meter and precision pH strips with 0.2 scale (pH ≈4.4). Radiochemical purity was assessed by iTLC using silica gel strips developed in a methanol/5 M ammonium acetate (3:1) mobile phase. The strips were analyzed using a linear TLC scanner, and all batches met the acceptance criterion of >90% radiochemical purity.

***In vivo* PET imaging and organ biodistribution**

Male BALB/cJRr mice (8 weeks old, 16-23 g, Janvier Labs) were housed in Tecniplast GM 900 IVC cages under controlled environmental conditions (12:12 h light/dark cycle, 19-23 °C, 12-15 air changes/hour) with ad libitum access to irradiated Picolab 5053 diet and filtered, autoclaved tap water, in accordance with ethical approval (FOTS ID 30696, Norway). *In vivo* PET imaging and *ex vivo* organ biodistribution studies were performed for the (*S)*-, (*R*)-, and (*S/R*)-enantiomers of [^68^Ga]Ga-FAPI-46 (n=12 per enantiomer group), with each mouse receiving an intravenous bolus injection via the tail vein of 4-5 MBq of radiotracer in a total volume of 100-200 µL. Prior to injection, animals were warmed on a heating pad (37°C) and anesthetized using 4% isoflurane in 100% O₂ for induction, followed by maintenance at 2.0-2.5% isoflurane during handling and imaging. Injection syringes were calibrated using a CRC-55t dose calibrator, and residual radioactivity was measured post-injection, decay-corrected to the time of administration, and subtracted to determine the net injected dose. All animals were monitored for adverse signs throughout the study. Mice in the 240-minute cohort (n=3 per enantiomer) were cannulated with a preclinical catheter (PC 30, 8PE10, pre-filled with 66 µL saline). Immediately after catheter flushing with saline, a 62-minute dynamic whole-body PET acquisition was performed, followed by a CT scan for attenuation correction and anatomical reference under anesthesia with continuous temperature monitoring (480 projections, 70 kVp tube voltage, 300 ms exposure time, 1:4 binning, and helical acquisition). After imaging, these animals were kept for an additional 3 hours before re-anesthesia and sacrifice. All animals were euthanized at their scheduled time points (n=3 per enantiomer per timepoint) for *ex vivo* biodistribution analysis.

**PET-imaging reconstruction and analysis**

The PET scan protocol was set to 62 minutes, with the first 2 minutes sorted into two 60-second frames (to accommodate injection timing), followed by 60 minutes divided into 19 frames: 4 x 15 s, 4 x 60 s, and 11 x 300 s. Image reconstruction was performed using whole-body 3D reconstruction with 2 iterations, 16 subsets, a 400–600 keV energy window, and a voxel size of 0.28 mm. Reconstructed images were analyzed using Imalytics software (version 3.1.1.0), and the percentage of injected activity per gram of tissue (%IA/g) was calculated using the formula:

$$\%IA/g tissue=\frac{x \left( MBq/g \right)}{\mathrm{IA}\left( \mathrm{MBq} \right)}\times100$$

Volumes of interest (VOIs) were defined for each animal, with brain, heart, liver, kidneys, and muscle segmented on CT images. Bladder activity was quantified using a PET image summed across all 21 time frames acquired over the 62-minute scan duration (2 × 60 s, 4 × 15 s, 4 × 60 s, and 11 × 300 s), providing an integrated measure of tracer accumulation.

***Ex vivo* organ biodistribution**

All excised organs and tissues were assayed for ^68^Ga-radioactivity using a Hidex AMG automatic gamma counter with a counting window of 480-558 keV. Each sample was counted for 30 seconds, and radioactivity was normalized to the time of the first sample counted. For organs with high activity, gamma counting was repeated later in the day to ensure the dead time factor was below 1.4. Bone marrow activity was assessed by comparing %IA in intact femur versus flushed marrow. For all other organs, absolute activity (MBq) was calculated from normalized counts (cps), corrected for detector efficiency using calibration-derived regression coefficients, and decay-corrected to the time of injection. The final %IA/g values were calculated using the same formula as for PET analysis.

**Supplementary Figures**

**Supplementary Figure 1.** **Stability of different enantiomeric compositions of [^68^Ga]Ga-FAPI-46 in human serum.** Radio-HPLC chromatograms of [^68^Ga]Ga-FAPI-46 in human serum over time. One representative experiment out of four is shown. Summary data are depicted in Figure 1A.

**Supplementary Figure 2.** **Stability of different enantiomeric compositions of [^68^Ga]Ga-FAPI-74 in human serum.** Radio-HPLC chromatograms of [^68^Ga]Ga-FAPI-74 in human serum over time. One representative experiment out of three is shown. Summary data are depicted in Figure 1B.

**
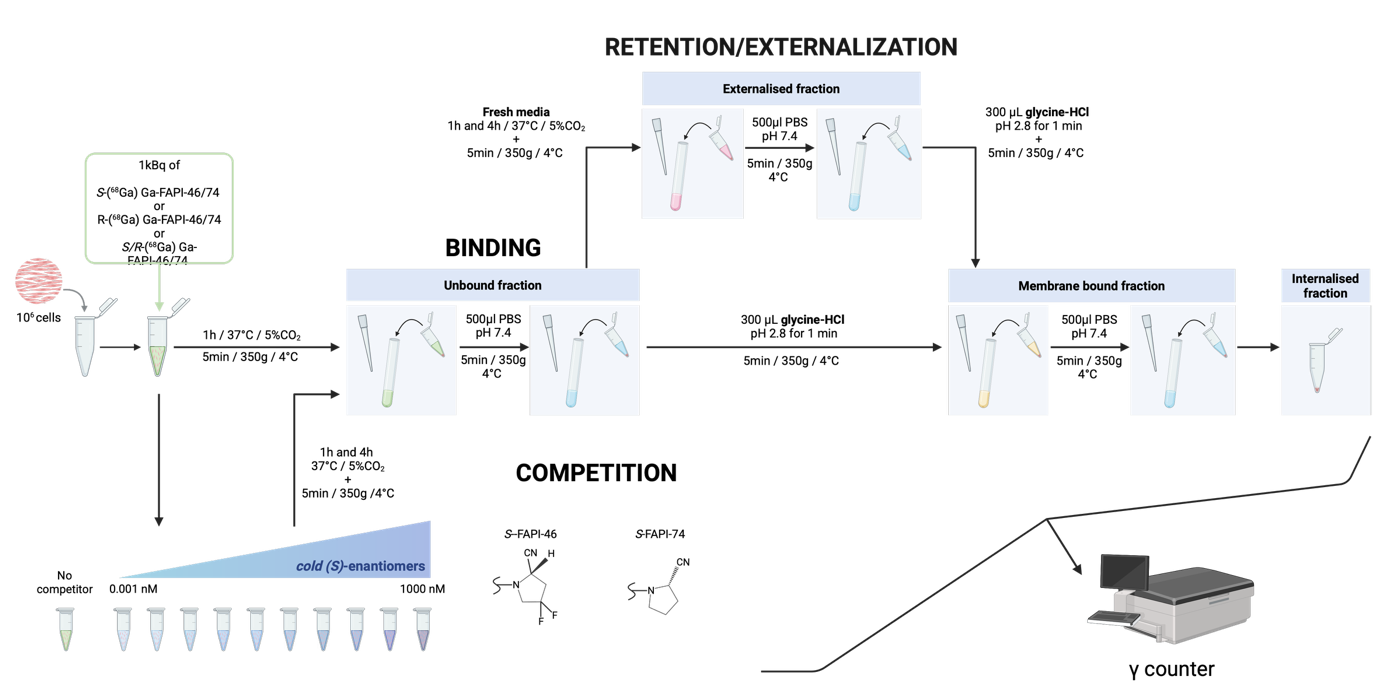
**

**Supplementary Figure 3.** Schematic illustrating the experimental workflow used to assess binding, internalization, and retention of different enantiomeric compositions in FAP-expressing cell lines. (Created in BioRender. https://BioRender.com/u8cydp6)


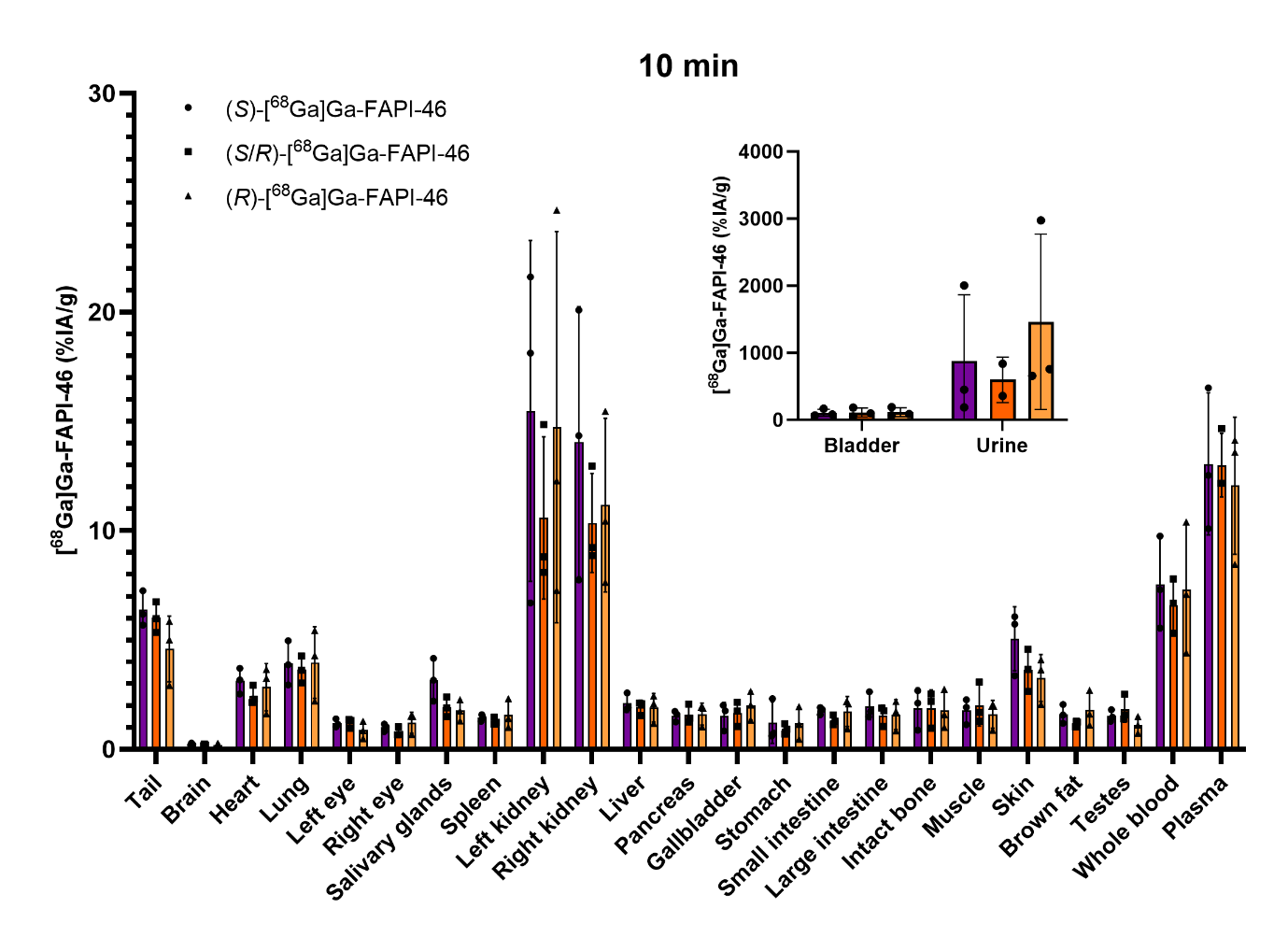
 **Supplementary Figure 4**. *Ex vivo* biodistribution of *(S)*-[^68^Ga]Ga-FAPI-46 (purple), *(S/R)*-[^68^Ga]Ga-FAPI-46 (orange), and *(R)*-[^68^Ga]Ga-FAPI-46 (yellow) in naïve mice (n=3/group) 10 min p.i. Differences in organ retention across the three enantiomeric compositions were not statistically significant, except for the following comparisons: left kidney: *(S)* vs. *(S/R)* p=0.0095, *(S/R)* vs. *(R)* p=0.0332.


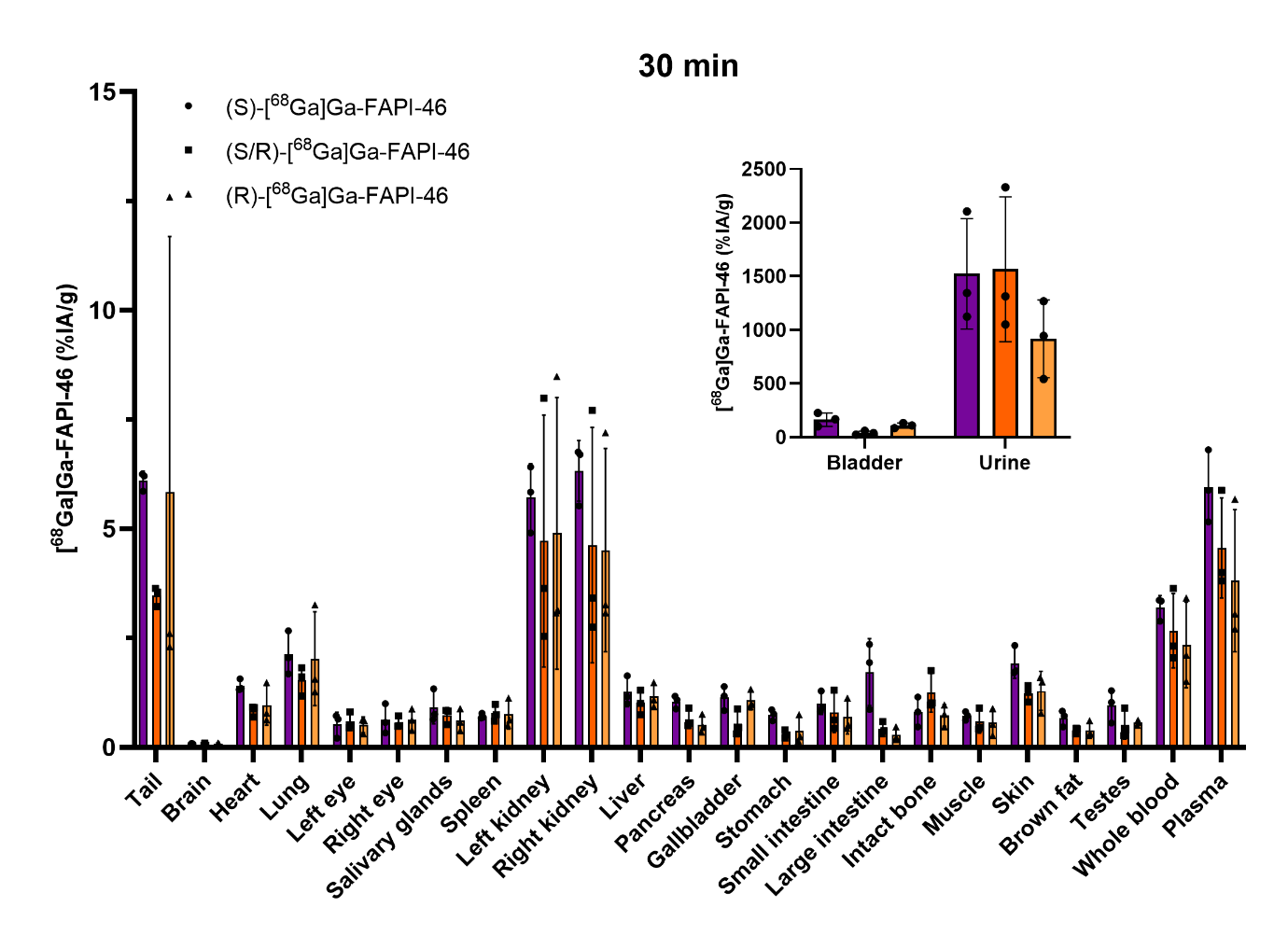


**Supplementary Figure 5.** *Ex vivo* biodistribution of *(S)*-[^68^Ga]Ga-FAPI-46 (purple), *(S/R)*-[^68^Ga]Ga-FAPI-46 (orange), and *(R)*-[^68^Ga]Ga-FAPI-46 (yellow) in naïve mice (n=3/group) 30 min p.i. Differences in organ retention across the three enantiomeric compositions were not statistically significant, except for the following comparisons: tail: *(S)* vs. *(S/R)* p=0.0080, *(S/R)* vs. *(R)* p=0.0198; plasma: *(S)* vs. *(R)* p=0.0397.


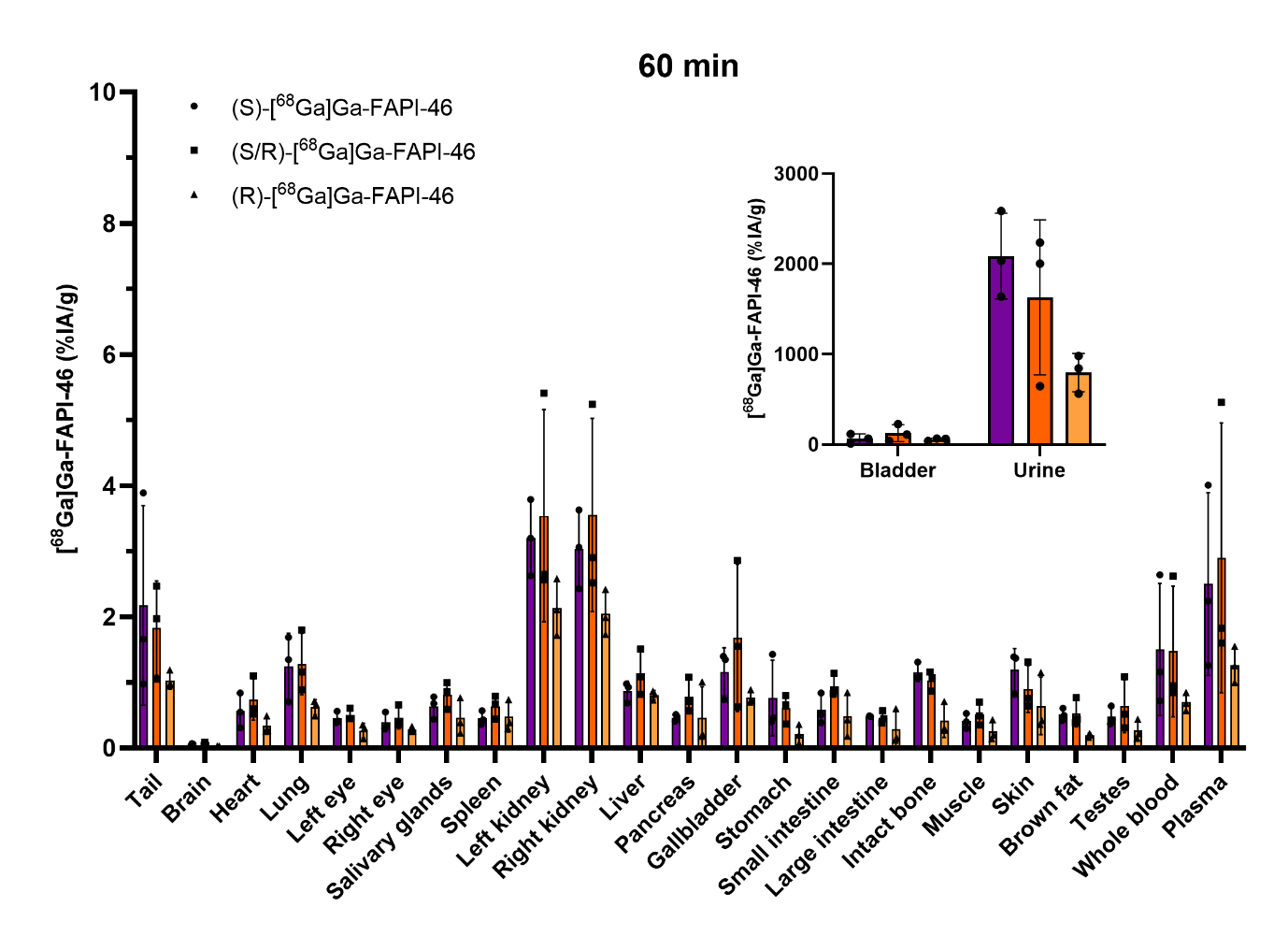
 **Supplementary Figure 6.** *Ex vivo* biodistribution of *(S)*-[^68^Ga]Ga-FAPI-46 (purple), *(S/R)*-[^68^Ga]Ga-FAPI-46 (orange), and *(R)*-[^68^Ga]Ga-FAPI-46 (yellow) in naïve mice (n=3/group) 60 min p.i. Differences in organ retention across the three enantiomeric compositions were not statistically significant, except for the following comparisons: tail: *(S)* vs. *(R)* p=0.0342; left kidney: *(S/R)* vs. *(R)* p=0.0066; right kidney: *(S/R)* vs. *(R)* p=0.0033; plasma: *(S)* vs. *(R)* p=0.0197, *(S/R)* vs. *(R)* p=0.0013; urine: *(S)* vs. *(R)* p=0063.


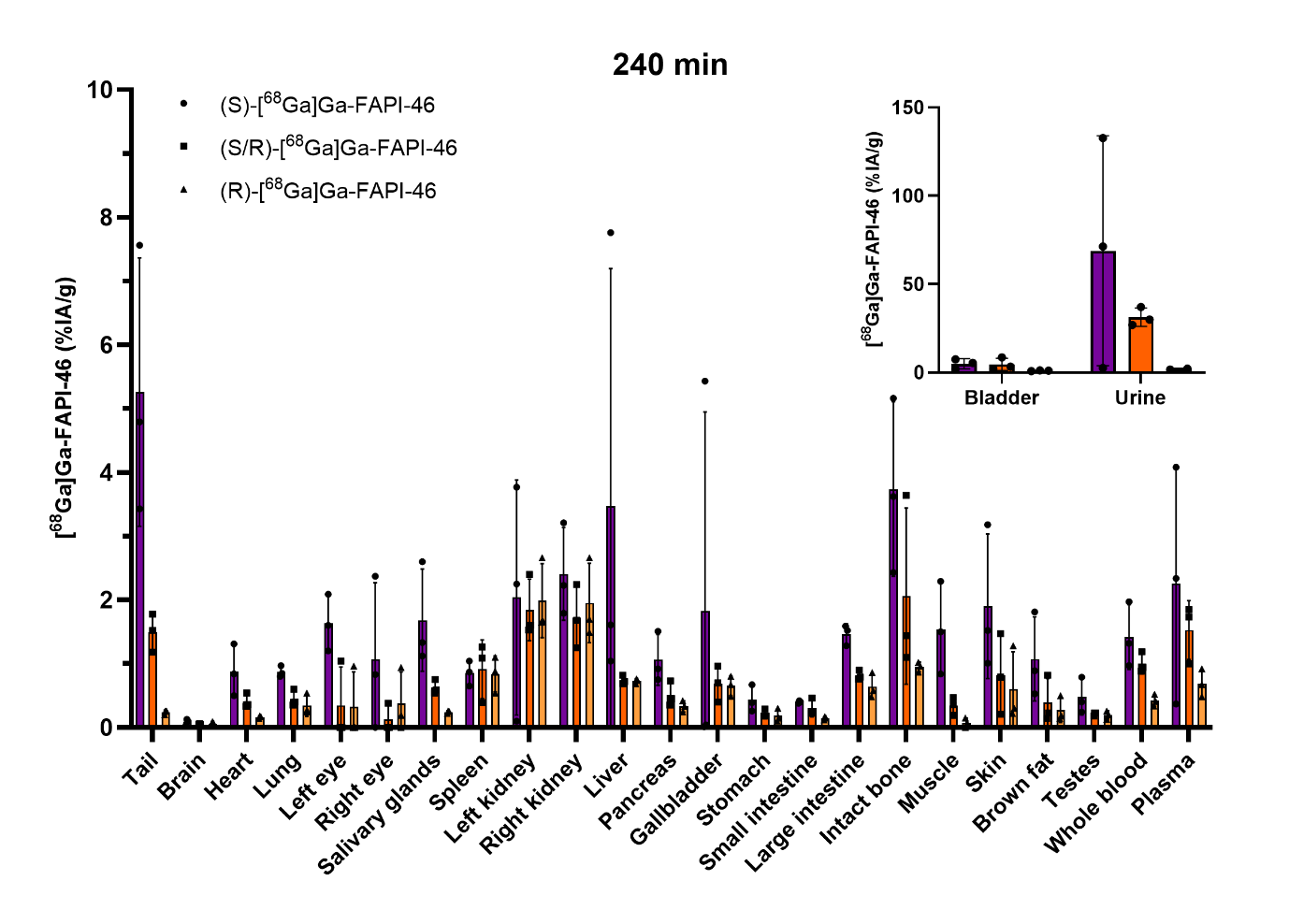


**Supplementary Figure 7.** *Ex vivo* biodistribution of *(S)*-[^68^Ga]Ga-FAPI-46 (purple), *(S/R)*-[^68^Ga]Ga-FAPI-46 (orange), and *(R)*-[^68^Ga]GaFAPI-46 (yellow) in naïve mice (n=3/group) 240 min p.i. Differences in organ retention across the three enantiomeric compositions were not statistically significant, except for the following comparisons: tail: *(S)* vs. *(S/R)* p=<0.0001, *(S)* vs. *(R)* p<0.0001; liver: *(S)* vs. *(S/R)* p=0.0003, *(S)* vs. *(R)* p=0.0003; intact bone: *(S)* vs. *(S/R)* p=0.0413, *(S)* vs. *(R)* p=0002.

**Supplementary Tables**

**Supplementary Table 1.** **Adhesion of serum proteins to different enantiomeric compositions of [^68^Ga]Ga-FAPI-46.** The mean ± SD of the fraction of [^68^Ga]Ga-FAPI-46 bound to proteins in human serum are given (n=4).

| **Time point** | ***(S)*-[^68^Ga]Ga-FAPI-46** | ***(S/R)*-[^68^Ga]Ga-FAPI-46** | ***(R)*-[^68^Ga]Ga-FAPI-46** |
| --- | --- | --- | --- |
| 10 min | 2.9 ± 2.2 % | 2.4 ± 1.3 % | 3.0 ± 1.8 % |
| 30 min | 3.3 ± 1.9 % | 3.4 ± 2.0 % | 3.8 ± 2.5 % |
| 60 min | 3.6 ± 1.5 % | 2.3 ± 0.9 % | 3.3 ± 1.6 % |
| 90 min | 3.7 ± 1.0 % | 4.8 ± 2.6 % | 3.8 ± 2.6 % |
| 120 min | 2.1 ± 2.4 % | 2.3 ± 2.2 % | 1.6 ± 1.7 % |
| 180 min | 3.1 ± 4.2 % | 2.7 ± 3.8 % | 1.7 ± 2.5 % |
| 240 min | 1.4 ± 3.1 % | 1.6 ± 3.5 % | 1.5 ± 3.5 % |

**Supplementary Table 2.** **Adhesion of serum proteins to different enantiomeric compositions of [^68^Ga]Ga-FAPI-74.** The mean ± SD of the fraction of [^68^Ga]Ga-FAPI-74 bound to proteins in human serum are given (n=3).

| **Time point** | ***(S)*-[^68^Ga]Ga-FAPI-74** | ***(S/R)*-[^68^Ga]Ga-FAPI-74** | ***(R)*-[^68^Ga]Ga-FAPI-74** |
| --- | --- | --- | --- |
| 10 min | 2.2 ± 1.2 % | 2.5 ± 1.8 % | 2.3 ± 3.3 % |
| 30 min | 1.3 ± 1.2 % | 2.5 ± 2.2 % | 2.4 ± 2.5 % |
| 60 min | 2.4 ± 1.2 % | 3.5 ± 1.3 % | 1.5 ± 1.3 % |
| 90 min | 2.2 ± 1.2 % | 2.4 ± 2.2 % | 4.2 ± 2.3 % |
| 120 min | 3.2 ± 2.1 % | 2.7 ± 2.6 % | 2.6 ± 2.3 % |
| 180 min | 1.2 ± 2.4 % | 3.2 ± 2.4 % | 3.4 ± 3.2 % |
| 240 min | 2.1 ± 2.1 % | 3.2 ± 3.4 % | 2.5 ± 2.3 % |

**Supplementary Table 3.** **Binding characteristics of different enantiomeric compositions of [^68^Ga]Ga-FAPI-46.** HT1080-hFAP and HT1080 cells were incubated with (*S*)-, (*S/R*)-, or (*R*)-[^68^Ga]Ga-FAPI-46 for 1 h or 4 h. The mean ± SD of the overall cell-associated and internalized fractions of [^68^Ga]Ga-FAPI-46 are given as % added activity per 10^6^ cells (%AA/10^6^ cells) (HT1080-hFAP: 1h, n=5, 4h, n=3; HT1080: n=6). P-values were determined using the Mann Whitney test and compare (*S*)-[^68^Ga]Ga-FAPI-46 and (*S/R*)-[^68^Ga]Ga-FAPI-46 binding characteristics on HT1080-hFAP cells to support conclusions regarding comparability. Data correspond to the summary data shown in Figure 2B,D.

|  | **HT1080-hFAP** | | | | | | **HT1080** | | | |
| --- | --- | --- | --- | --- | --- | --- | --- | --- | --- | --- |
|  | **1 h** | | | **4 h** | | | **1 h** | | **4 h** | |
|  | **B** | **I** | **%I** | **B** | **I** | **%I** | **B** | **I** | **B** | **I** |
| ***(R)*** | 2.3 ± 0.7 | 1.6 ± 0.8 |  | 4.3 ± 2.31 | 2.8 ± 1.3 |  | 2.5 ± 0.8 | 1.7 ± 0.6 | 4.4 ± 2.3 | 3.0 ± 1.2 |
| ***(S)*** | 29.5 ± 12.7 | 23.8 ± 10.7 | 80.82 | 33.9 ± 4.8 | 26.6 ± 4.2 | 78.56 | 3.0 ± 1.6 | 2.4 ± 1.2 | 5.5 ± 3.8 | 3.3 ± 1.5 |
| ***(S/R)*** | 24.2 ± 9.5 (p=0.4206) | 19.4 ± 7.9  (p=0.4206) | 80.36 | 25.6 ± 2.3 (p=0.1000) | 20.6 ± 3.5 (p=0.2000) | 80.39 | 3.2 ± 1.1 | 2.2 ± 1.1 | 5.5 ± 2.6 | 3.6 ± 1.7 |

B – bound, overall cell-associated fraction; I – internalized fraction; %I – internalized fraction as percentage of overall cell-associated activity; (*R*) - (*R*)-[^68^Ga]Ga-FAPI-46, (*S*) - (*S*)-[^68^Ga]Ga-FAPI-46, (S/*R*) - (S/*R*)-[^68^Ga]Ga-FAPI-46

**Supplementary Table 4.** **Binding characteristics of different enantiomeric compositions of [^68^Ga]Ga-FAPI-74.** HT1080-hFAP and HT1080 cells were incubated with (*S*)-, (*S/R*)-, or (*R*)-[^68^Ga]Ga-FAPI-74 for 1 h or 4 h. The mean ± SD of the overall cell-associated and internalized fractions of [^68^Ga]Ga-FAPI-74 are given as % added activity per 10^6^ cells (%AA/10^6^ cells) (n=3). P-values were determined using the Mann Whitney test and compare (*S*)-[^68^Ga]Ga-FAPI-74 and (*S/R*)-[^68^Ga]Ga-FAPI-74 binding characteristics on HT1080-hFAP cells to support conclusions regarding comparability. Data correspond to the summary data shown in Figure 2C,E.

|  | **HT1080-hFAP** | | | | | |
| --- | --- | --- | --- | --- | --- | --- |
|  | **1 h** | | | **4 h** | | |
|  | **B** | **I** | **%I** | **B** | **I** | **%I** |
| ***(R)*** | 1.0 ± 0.5 | 0.6 ± 0.6 |  | 4.5 ± 2.1 | 3.9 ± 2.5 |  |
| ***(S)*** | 26.2 ± 5.6 | 20.1 ± 5.2 | 76.6 | 19.4 ± 1.4 | 11.7 ± 2.6 | 60.0 |
| ***(S/R)*** | 23.4 ± 2.1 (p=0.7000) | 19.2 ± 1.4 (p=0.7000) | 82.0 | 19.2 ± 2.8 (p>0.9999) | 12.8 ± 2.1 (p>0.9999) | 66.70 |

|  | **HT1080** | | | |
| --- | --- | --- | --- | --- |
|  | **1 h** | | **4 h** | |
|  | **B** | **I** | **B** | **I** |
| ***(R)*** | 1.9 ± 1.9 | 1.1 ± 1.2 | 4.9 ± 2.6 | 1.4 ± 0.8 |
| ***(S)*** | 1.7 ± 1.3 | 0.8 ± 0.7 | 4.5 ± 3.3 | 1.6 ± 1.3 |
| ***(S/R)*** | 1.6 ± 1.6 | 0.9 ± 1.5 | 3.8 ± 4.0 | 1.1 ± 0.6 |

B – bound, overall cell-associated fraction; I – internalized fraction; %I – internalized fraction as percentage of overall cell-associated activity; (*R*) - (*R*)-[^68^Ga]Ga-FAPI-74, (*S*) - (*S*)-[^68^Ga]Ga-FAPI-74, (S/*R*) - (S/*R*)-[^68^Ga]Ga-FAPI-74

**Supplementary Table 5. Retention of different enantiomeric compositions of [^68^Ga]Ga-FAPI-46.** HT1080-hFAP cells were incubated with the (*S*)-, or (*S/R*)-[^68^Ga]Ga-FAPI-46 for 1 h. Cells were washed, incubated with non-radioactive medium for 1 h or 4 h, and internalized fractions were analyzed. The mean ± SD of the initially cell-associated activity (B) is given as % added activity per 10^6^ cells (%AA/10^6^ cells); the radioligand fraction that was still internalized (retained) after 1 h and 4 h is given as percent of the initially cell-associated activity (n=4). P-values were determined using the Mann Whitney test and compare (*S*)-[^68^Ga]Ga-FAPI-46 and (*S/R*)-[^68^Ga]Ga-FAPI-46 to support conclusions regarding comparability. Data correspond to the summary data shown in Figure 2H.

| **HT1080-hFAP** | | | | |
| --- | --- | --- | --- | --- |
|  | **1 h** | | **4 h** | |
|  | **B, t = 0** | **%I, t = 1 h** | **B, t = 0** | **%I, t = 4 h** |
| ***(S)*** | 38.8 ± 4.7 | 54.3 ± 7.3 | 40.3 ± 3.8 | 45.9 ± 11.3 |
| ***(S/R)*** | 36.2 ± 4.5 (p=0.8571) | 53.8 ± 7.0 (p>0.9999) | 36.4 ± 7.1 (p=0.3943) | 34.7 ± 4.0 (p=0.1143) |

B – bound, overall cell-associated fraction after the 1 h incubation with radioligand (t=0); %I – activity that is retained in the cell after 1 h and 4 h in non-radioactive media as percent of initially (t=0) cell-associated fraction; (*S*) - (*S*)-[^68^Ga]Ga-FAPI-46, (S/*R*) - (S/*R*)-[^68^Ga]Ga-FAPI-46

**Supplementary Table 6. Retention of different enantiomeric compositions of [^68^Ga]Ga-FAPI-74.** HT1080-hFAP cells were incubated with the (*S*)-, or (*S/R*)-[^68^Ga]Ga-FAPI-74 for 1 h. Cells were washed, incubated with non-radioactive medium for 1 h, and internalized fractions were analyzed. The mean ± SD of the initially cell-associated activity (B) is given as % added activity per 10^6^ cells (%AA/10^6^ cells); the radioligand fraction that was still internalized (retained) after 1 h is given as percent of the initially cell-associated activity (n=3). P-values were determined using the Mann Whitney test and compare (*S*)-[^68^Ga]Ga-FAPI-74 and (*S/R*)-[^68^Ga]Ga-FAPI-74 to support conclusions regarding comparability. Data correspond to the summary data shown in Figure 2I.

|  | **HT1080-hFAP** | |
| --- | --- | --- |
|  | **B, t = 0** | **%I, t = 1 h** |
| ***(S)*** | 25.9 ± 3.7 | 43.7 ± 8.7 |
| ***(S/R)*** | 25.8 ± 5.2  (p>0.9999) | 34.8 ± 15.8 (p=0.7000) |

B – bound, overall cell-associated fraction after the 1 h incubation with radioligand; %I – activity that is retained in the cell after 1 h and 4 h in non-radioactive media as percent of initially (t=0) cell-associated fraction; (*S*) - (*S*)-[^68^Ga]Ga-FAPI-74, (S/*R*) - (S/*R*)-[^68^Ga]Ga-FAPI-74

**Supplementary Table 7. Biodistribution of *(S)*-[^68^Ga]FAPI-46.** Biodistribution data are presented as mean %IA/g tissue at 10, 30, 60 and 240 min post-injection of 4-5 MBq *(S)*-[^68^Ga]Ga-FAPI-46 into the tail vein of Balb/c mice (n=3/group).

| **%IA/g tissue** | | | | | | | | |
| --- | --- | --- | --- | --- | --- | --- | --- | --- |
|  | *10 min* | | *30 min* | | *60 min* | | *240 min* | |
| *Organ* | *Mean* | *SD* | *Mean* | *SD* | *Mean* | *SD* | *Mean* | *SD* |
| Tail | 6.38 | 0.66 | 6.11 | 0.17 | 2.18 | 1.24 | 5.26 | 1.72 |
| Brain | 0.23 | 0.03 | 0.08 | 0.01 | 0.06 | 0.02 | 0.09 | 0.04 |
| Heart | 3.14 | 0.48 | 1.42 | 0.11 | 0.57 | 0.22 | 0.88 | 0.33 |
| Lung | 3.93 | 0.83 | 2.13 | 0.41 | 1.25 | 0.41 | 0.87 | 0.07 |
| Left eye | 1.19 | 0.16 | 0.53 | 0.23 | 0.45 | 0.08 | 1.63 | 0.36 |
| Right eye | 1.00 | 0.15 | 0.63 | 0.28 | 0.39 | 0.11 | 1.06 | 0.98 |
| Salivary glands | 3.17 | 0.80 | 0.91 | 0.31 | 0.63 | 0.14 | 1.68 | 0.65 |
| Spleen | 1.44 | 0.13 | 0.71 | 0.05 | 0.45 | 0.08 | 0.85 | 0.16 |
| Left kidney | 15.47 | 6.37 | 5.72 | 0.62 | 3.21 | 0.48 | 2.04 | 1.51 |
| Right kidney | 14.06 | 5.05 | 6.33 | 0.57 | 3.04 | 0.49 | 2.41 | 0.59 |
| Liver | 2.10 | 0.35 | 1.27 | 0.27 | 0.87 | 0.13 | 3.47 | 3.04 |
| Pancreas | 1.53 | 0.20 | 1.05 | 0.12 | 0.46 | 0.04 | 1.06 | 0.33 |
| Gallbladder | 1.54 | 0.50 | 1.14 | 0.23 | 1.16 | 0.30 | 1.82 | 2.55 |
| Stomach | 1.22 | 0.78 | 0.75 | 0.11 | 0.77 | 0.47 | 0.44 | 0.17 |
| Small intestine | 1.77 | 0.14 | 1.01 | 0.21 | 0.58 | 0.19 | 0.40 | 0.02 |
| Large intestine | 1.95 | 0.49 | 1.72 | 0.63 | 0.48 | 0.00 | 1.46 | 0.13 |
| Intact bone | 1.89 | 0.75 | 0.81 | 0.28 | 1.15 | 0.11 | 3.74 | 1.12 |
| Muscle | 1.78 | 0.48 | 0.72 | 0.09 | 0.41 | 0.09 | 1.54 | 0.59 |
| Skin | 5.06 | 1.20 | 1.93 | 0.28 | 1.19 | 0.26 | 1.90 | 0.93 |
| Brown fat | 1.61 | 0.35 | 0.67 | 0.15 | 0.51 | 0.08 | 1.08 | 0.54 |
| Bladder | 105.77 | 43.60 | 164.39 | 51.17 | 64.34 | 43.45 | 4.93 | 2.30 |
| Testes | 1.52 | 0.22 | 0.96 | 0.31 | 0.48 | 0.12 | 0.48 | 0.23 |
| Whole blood | 7.54 | 1.73 | 3.20 | 0.22 | 1.51 | 0.82 | 1.42 | 0.42 |
| Plasma | 13.05 | 2.66 | 5.95 | 0.68 | 2.50 | 1.14 | 2.26 | 1.51 |
| Urine | 881.67 | 802.02 | 1524.79 | 420.08 | 2085.98 | 388.48 | 101.97 | 30.72 |

**Supplementary Table 8. Biodistribution of *(S/R)*-[^68^Ga]Ga-FAPI-46.** Biodistribution data are presented as mean %IA/g tissue at 10, 30, 60 and 240 min post-injection of 4-5 MBq *(S/R)*-[^68^Ga]Ga-FAPI-46 into the tail vein of BALB/c mice (n=3/group).

| **%IA/g tissue** | | | | | | | | |
| --- | --- | --- | --- | --- | --- | --- | --- | --- |
|  | *10 min* | | *30 min* | | *60 min* | | *240 min* | |
| *Organ* | *Mean* | *SD* | *Mean* | *SD* | *Mean* | *SD* | *Mean* | *SD* |
| Tail | 6.03 | 0.57 | 3.47 | 0.18 | 1.83 | 0.59 | 1.49 | 0.24 |
| Brain | 0.20 | 0.03 | 0.06 | 0.02 | 0.05 | 0.02 | 0.05 | 0.00 |
| Heart | 2.46 | 0.35 | 0.84 | 0.10 | 0.74 | 0.25 | 0.41 | 0.09 |
| Lung | 3.64 | 0.50 | 1.54 | 0.27 | 1.28 | 0.38 | 0.45 | 0.11 |
| Left eye | 1.21 | 0.18 | 0.59 | 0.16 | 0.51 | 0.08 | 0.35 | 0.49 |
| Right eye | 0.79 | 0.17 | 0.56 | 0.11 | 0.46 | 0.14 | 0.13 | 0.18 |
| Salivary glands | 1.93 | 0.37 | 0.73 | 0.15 | 0.82 | 0.17 | 0.63 | 0.09 |
| Spleen | 1.28 | 0.14 | 0.78 | 0.16 | 0.64 | 0.15 | 0.91 | 0.37 |
| Left kidney | 10.59 | 3.04 | 4.72 | 2.35 | 3.54 | 1.32 | 1.84 | 0.39 |
| Right kidney | 10.35 | 1.85 | 4.63 | 2.20 | 3.55 | 1.20 | 1.72 | 0.40 |
| Liver | 1.92 | 0.26 | 1.02 | 0.23 | 1.14 | 0.29 | 0.75 | 0.06 |
| Pancreas | 1.57 | 0.35 | 0.65 | 0.18 | 0.78 | 0.21 | 0.51 | 0.16 |
| Gallbladder | 1.64 | 0.46 | 0.55 | 0.24 | 1.68 | 0.92 | 0.69 | 0.23 |
| Stomach | 0.93 | 0.19 | 0.30 | 0.07 | 0.61 | 0.18 | 0.21 | 0.05 |
| Small intestine | 1.33 | 0.18 | 0.79 | 0.37 | 0.95 | 0.14 | 0.30 | 0.11 |
| Large intestine | 1.56 | 0.37 | 0.43 | 0.12 | 0.47 | 0.08 | 0.82 | 0.06 |
| Intact bone | 1.89 | 0.67 | 1.25 | 0.36 | 1.03 | 0.12 | 2.06 | 1.13 |
| Muscle | 2.01 | 0.77 | 0.60 | 0.22 | 0.52 | 0.14 | 0.34 | 0.12 |
| Skin | 3.62 | 0.79 | 1.23 | 0.15 | 0.91 | 0.29 | 0.82 | 0.51 |
| Brown fat | 1.18 | 0.11 | 0.39 | 0.06 | 0.53 | 0.17 | 0.40 | 0.30 |
| Bladder | 111.78 | 54.13 | 39.90 | 14.51 | 125.40 | 77.04 | 4.65 | 2.79 |
| Testes | 1.84 | 0.49 | 0.52 | 0.27 | 0.64 | 0.33 | 0.21 | 0.03 |
| Whole blood | 6.60 | 1.02 | 2.67 | 0.69 | 1.47 | 0.81 | 1.00 | 0.13 |
| Plasma | 13.00 | 1.18 | 4.56 | 0.94 | 2.90 | 1.68 | 1.53 | 0.38 |
| Urine | 598.75 | 240.69 | 1565.46 | 551.81 | 1629.14 | 700.95 | 31.31 | 4.25 |

**Supplementary Table 9. Biodistribution of *(R)*-[^68^Ga]GaFAPI-46.** Biodistribution data are presented as mean %IA/g tissue at 10, 30, 60 and 240 min post-injection of 4-5 MBq *(R)*-[^68^Ga]Ga-FAPI-46 into the tail vein of BALB/c mice (n=3).

| **%IA/g tissue** | | | | | | | | |
| --- | --- | --- | --- | --- | --- | --- | --- | --- |
|  | *10 min* | | *30 min* | | *60 min* | | *240 min* | |
| *Organ* | *Mean* | *SD* | *Mean* | *SD* | *Mean* | *SD* | *Mean* | *SD* |
| Tail | 4.59 | 1.23 | 5.83 | 4.78 | 1.03 | 0.11 | 0.24 | 0.03 |
| Brain | 0.19 | 0.06 | 0.07 | 0.02 | 0.03 | 0.00 | 0.06 | 0.03 |
| Heart | 2.84 | 0.88 | 0.96 | 0.37 | 0.34 | 0.11 | 0.16 | 0.02 |
| Lung | 3.97 | 1.34 | 2.03 | 0.88 | 0.62 | 0.10 | 0.34 | 0.14 |
| Left eye | 0.87 | 0.32 | 0.52 | 0.15 | 0.27 | 0.09 | 0.32 | 0.45 |
| Right eye | 1.22 | 0.39 | 0.63 | 0.20 | 0.28 | 0.04 | 0.38 | 0.40 |
| Salivary glands | 1.78 | 0.40 | 0.61 | 0.21 | 0.47 | 0.23 | 0.24 | 0.02 |
| Spleen | 1.57 | 0.55 | 0.76 | 0.27 | 0.48 | 0.19 | 0.84 | 0.23 |
| Left kidney | 14.74 | 7.32 | 4.90 | 2.54 | 2.13 | 0.35 | 1.99 | 0.48 |
| Right kidney | 11.18 | 3.24 | 4.51 | 1.90 | 2.05 | 0.28 | 1.95 | 0.51 |
| Liver | 1.90 | 0.53 | 1.17 | 0.23 | 0.81 | 0.06 | 0.73 | 0.03 |
| Pancreas | 1.61 | 0.41 | 0.52 | 0.17 | 0.46 | 0.39 | 0.33 | 0.07 |
| Gallbladder | 2.00 | 0.54 | 1.08 | 0.18 | 0.77 | 0.09 | 0.65 | 0.13 |
| Stomach | 1.19 | 0.61 | 0.38 | 0.26 | 0.21 | 0.12 | 0.18 | 0.08 |
| Small intestine | 1.73 | 0.56 | 0.69 | 0.31 | 0.49 | 0.28 | 0.15 | 0.02 |
| Large intestine | 1.59 | 0.56 | 0.28 | 0.14 | 0.29 | 0.22 | 0.64 | 0.16 |
| Intact bone | 1.78 | 0.73 | 0.74 | 0.20 | 0.42 | 0.21 | 0.95 | 0.06 |
| Muscle | 1.61 | 0.52 | 0.56 | 0.25 | 0.26 | 0.12 | 0.07 | 0.06 |
| Skin | 3.26 | 0.88 | 1.29 | 0.36 | 0.64 | 0.36 | 0.60 | 0.49 |
| Brown fat | 1.80 | 0.67 | 0.39 | 0.15 | 0.19 | 0.02 | 0.27 | 0.16 |
| Bladder | 115.99 | 54.32 | 108.83 | 19.77 | 56.46 | 12.77 | 0.99 | 0.17 |
| Testes | 1.11 | 0.31 | 0.57 | 0.05 | 0.27 | 0.13 | 0.20 | 0.05 |
| Whole blood | 7.30 | 2.45 | 2.35 | 0.80 | 0.70 | 0.12 | 0.43 | 0.08 |
| Plasma | 12.06 | 2.57 | 3.82 | 1.33 | 1.27 | 0.23 | 0.69 | 0.18 |
| Urine | 1463.30 | 1069.56 | 917.75 | 296.56 | 796.61 | 173.91 | 1.92 | 0.27 |
